# The impact of fatigue on lower limb neuromuscular control in female college soccer players

**DOI:** 10.3389/fspor.2026.1918634

**Published:** 2026-10-16

**Authors:** Tao Xiang, Tingzheng Zhu, Huiru Ma, Ziwen Mu, Zhiqiang Han, Kazuhiro Imai, Shaoshuai Shen, Zhiguang Guo, Xiao Zhou

**Affiliations:** 1 School of Physical Education, Huazhong University of Science and Technology, Wuhan, China; 2 Department of Life Sciences, Graduate School of Arts and Sciences, The University of Tokyo, Tokyo, Japan; 3 School of Education and Welfare, Aichi Prefectural University, Nagakute, Japan; 4 School of Sports Health, Hubei University of Chinese Medicine, Wuhan, China

**Keywords:** fatigue, female players, lower limb muscle activation, neuromuscular control, soccer

## Abstract

**Introduction:**

Fatigue is a key risk factor for non-contact lower-limb injuries in female soccer players. This study aimed to identify the impact of fatigue on lower limb neuromuscular control in female college soccer players.

**Methods:**

Twenty female collegiate soccer players performed the Yo-Yo Intermittent Endurance Level 2 Test for fatigue induction. Before and after fatigue, single-leg landing (SLL), double-leg landing (DLL), and zigzag running were assessed using surface electromyography (sEMG) and force plates. Normalized sEMG amplitude (%PEAK), peak vertical ground reaction force (vGRF, BW), stabilization time, and center of pressure (COP) displacement area were analyzed. Twenty participants performed the Yo-Yo Intermittent Endurance Level 2 Test for fatigue induction. Before and after the test, single-leg landing (SLL), double-leg landing (DLL), zigzag running, surface electromyography, force plates, and center of pressure analysis were used to assess neuromuscular and kinetic responses. Fatigue altered sEMG amplitude in a task-specific manner.

**Results:**

In DLL, normalized sEMG amplitude decreased significantly in the right vastus lateralis (*P* = 0.002; FDR-adjusted *P* = 0.024), left vastus lateralis (*P* = 0.006; FDR-adjusted *P* = 0.036), and right vastus medialis (*P* = 0.010; FDR-adjusted *P* = 0.040). In zigzag running, a pre- fatigue bilateral rectus femoris asymmetry (*P* = 0.037; exploratory, FDR- adjusted *P* > 0.05) was no longer significant post-fatigue (*P* = 0.112). In SLL, peak vGRF (BW) decreased bilaterally (left: 2.52 ± 0.31–2.34 ± 0.27 BW, *P* = 0.015; FDR-adjusted *P* = 0.045; right: 2.69 ± 0.36-2.40 ± 0.30 BW, *P* < 0.001; FDR-adjusted *P* < 0.003).

**Discussion:**

These findings provide insights into fatigue-induced neuromuscular changes in female collegiate soccer players, with the DLL vastus muscles' responses surviving FDR correction, while other findings remain exploratory and require confirmation. The results offer preliminary theoretical support for targeted training and injury prevention strategies, though the fixed testing sequence and potential recovery effects limit strong causal conclusions.

## Introduction

As of 2023, the number of women participating in organized football worldwide has increased by nearly a quarter compared to 2019, reaching approximately 16.6 million (1). Parallel to this global trend, the collegiate sector has emerged as a vital framework for the expansion of women's soccer, witnessing a historical surge in female student-athlete participation worldwide (2).

Soccer is characterized by high intensity, frequent multidirectional changes, and sudden stops, creating a highly dynamic environment. Physical contact between players increases biomechanical loads on lower limb joints, exposing players to increase risk of soccer related injuries (3–5). Due to physiological and biomechanical differences, female players exhibit a significantly higher risk of specific lower-limb injuries, particularly non-contact anterior cruciate ligament (ACL) injuries, compared to male players (6, 7), with female soccer players sustaining 4–6 times the risk (8, 9). Moreover, increased valgus moment during landing and change-of-direction tasks is one of the primary risk factors for ACL injuries in female athletes (10, 11).

Importantly, a considerable number of these non-contact injuries occur after players experience fatigue, with fatigue, bilateral lower limb functional asymmetry, and neuromuscular control deficits identified as key risk factors (12, 13). During competition, collegiate women's soccer players experienced significant fatigue responses, manifested as immediate declines in neuromuscular indicators such as countermovement jump (CMJ) (14). This fatigue is typically closely associated with external loads (e.g., high-speed running distance) and internal loads (e.g., changes in heart rate) during competition (14, 15).

When fatigue occurs, timely monitoring of neuromuscular indicators is crucial for understanding injury mechanisms. Surface electromyography (sEMG) can capture changes in muscle activation timing and intensity, including neuromuscular control asymmetry between bilateral limbs (16), while force plates can detect asymmetries in dynamic balance ability that may increase injury risk (10, 17). Combining these technologies may provide insights into non-contact injury mechanisms in fatigued female soccer players.

Among female collegiate soccer players, past studies have indicated that their training loads and periodization can be less stable compared to professional players (18). Furthermore, training for collegiate athletes may focus more on enhancing fundamental strength and athletic performance (19) rather than the high-volume, sport-specific neuromuscular conditioning required for professional careers (20). These differences in training history and adaptation may predispose collegiate players to distinct neuromuscular control patterns and potentially higher-risk movement strategies under fatigue, a hypothesis that warrants further investigation.

Therefore, this study aimed to examine the kinetic changes in lower-limb among female college soccer players before and after fatigue induction using sEMG and force plate. We hypothesized that: (1) fatigue would lead to a significant decrease in sEMG amplitude, particularly in the knee extensor muscles (e.g., vastus lateralis and medialis) during landing tasks; and (2) pre-existing asymmetries in lower limb kinetic and EMG variables between the left and right sides would be exacerbated or significantly altered following fatigue induction.

## Materials and methods

### Participants

We recruited 21 female college soccer players aged 19–23 from two women's soccer teams at Chinese universities. Prior to the experiment, baseline data were recorded, including age, height, weight, years of training, training frequency, dominant foot, and lower limb injury history. Of these, one participant was excluded due to incomplete sEMG data resulting from a technical error during post-fatigue testing. Therefore, data from 20 participants were included in the final analysis. The experiment was performed from June 1 to October 12, 2025.

The inclusion criteria for participants were: (1) Aged 18 years or above, with no neuromuscular or skeletal disorders, based on self-report during the screening questionnaire; (2) Having participated in or currently participating in Chinese university football competitions; (3) Receiving at least three soccer training sessions per week for the past year or on a long-term basis. Exclusion criteria were: (1) Athletes who have engaged in strenuous exercise within 24 h prior to the experiment; (2) A history of lower limb injury in the past 6 months that required medical attention or resulted in time loss from training/competition; (3) Incomplete or missing experimental test data.

The sample size was estimated *a priori* using G-Power (version 3.1.9.7; Heinrich Heine University Düsseldorf, Germany). Based on previous studies reporting large effect sizes for fatigue-induced changes in neuromuscular variables (10, 21), an effect size of 0.8 was assumed. As our data included both normally and non-normally distributed variables, sample size was estimated separately for a paired *t*-test and its nonparametric counterpart (Wilcoxon signed-rank test). With a significance level of *α* = 0.05 and a desired power of 0.90, the required total sample sizes were 19 and 20, respectively.

Field positions were recorded during screening (defender: *n* = 8, midfielder: *n* = 6, forward: *n* = 4, goalkeeper: *n* = 2). However, position-specific analyses were not performed due to the small sample size and the exploratory nature of the study. All participants were informed of the study protocol and signed informed consent forms prior to the experiment as well as instructed to avoid strenuous exercise and to maintain their habitual sleep and dietary patterns for 24 h prior to the experiment. This research was approved by the Institutional Review Board of Huazhong University of Science and Technology [Approval No. [2023] IEC (S172)] and strictly adhered to the principles outlined in the 2013 revised Declaration of Helsinki.

### Fatigue induction

The Yo-Yo Intermittent Endurance Level 2 Test (Yo-YoIE2) with high validity and reliability serves as a general short-term fatigue induction protocol for soccer players, widely applied in both male and female athletes (22–25). The Yo-YoIE2 assesses athletes’ capacity for sustained intermittent work over extended periods, making it the fatigue induction protocol selected for this study.

The Yo-YoIE2 involves repeated 2 × 20-meter shuttle runs (totaling 40 meters per bout) between two marked lines (Figure 1), with progressively increasing speed controlled by a CD recording signal (25). Each sprint is separated by 5 seconds of active recovery, consisting of jogging within a 2.5-meter buffer zone. The test finishes when a subject fails to reach the line twice upon the recording signal, and the total distance completed was recorded. The test was conducted on an indoor track that was 1.5 meters wide and 22.5 meters long (with a 2.5-meter buffer zone), with cones placed at both ends; participants were required to wear athletic shoes.

**Figure 1:**
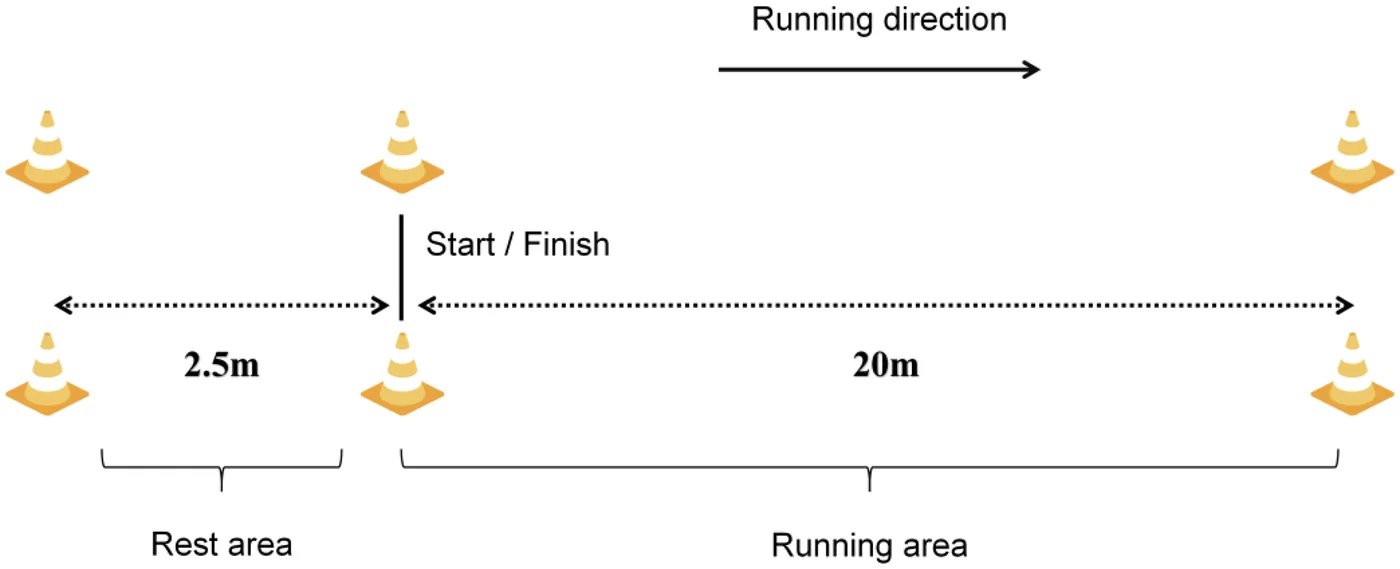
Yo-yo intermittent endurance level 2 test (yo-YoIE2).

Since fatigue is defined as a subjective sensation or an objective state (12, 13), and previous research has indicated that the relationship between objective fatigue variables (e.g., changes in jump height) and subjective fatigue is complex and not always linearly correlated, it is essential to explicitly define the assessment approach when characterizing fatigue (26). Thus, to assess fatigue levels during testing precisely, all participants wore heart rate monitors throughout the process and completed the Borg 6-20 Rating of Perceived Exertion (RPE) scale (often referred to as the 15-point scale) after fatigue induction (27, 28). Fatigue level was determined by meeting either of the following conditions: (1) Heart rate reached 85% of estimated maximum heart rate (220—age), a commonly used, albeit approximate, threshold in fatigue studies (21), where estimated maximum heart rate = 220—age; (2) RPE reached level 15 or higher. Heart rate was monitored in real-time for safety purposes and was not recorded for subsequent analysis. All tests were conducted in a laboratory environment with a controlled room temperature of approximately 25°C.

### Single-leg landing (SLL) and double-leg landing (DLL) tests

SLL and DLL tests are commonly used as models for studying landing biomechanics to evaluate lower limb injury risks (29).

In the SLL test (Figure 2a), participants were instructed to perform a fixed two-handed drop from a 30 cm platform, initiating each trial in a single-leg stance and landing in a fixed manner on the force plate on the same side. The dominant leg was defined as the leg the participant preferred to use for kicking a ball and could kick for maximum distance (30). In the DLL test (Figure 2B), participants performed a drop from a 30 cm platform, landing with both feet simultaneously, each on a separate force plate.

**Figure 2:**
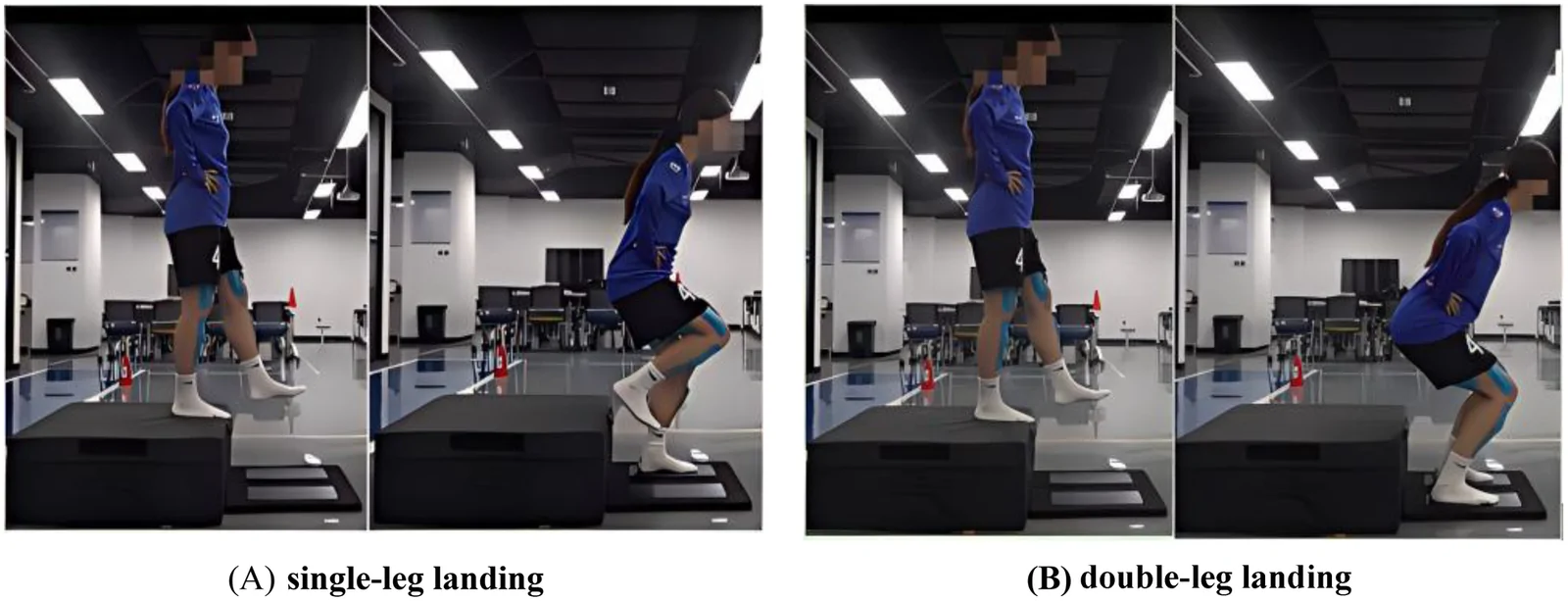
Two different landing tasks. **(A)** single-leg landing (SLL), **(B)** double-leg landing (DLL).

To control for potential effects of hand position on landing and neuromuscular dynamics, participants in all trials were instructed to maintain a forward gaze at horizontal level and keep hands on the upper hips at all times (29). Trials were repeated if participants lost balance, removed their hands from their hips at any point during the trial, or failed to land completely within the boundaries of the force plate (s) (29).

### Zigzag running

Zigzag running is a dynamic change-of-direction running technique requiring rapid directional shifts and center-of-gravity adjustments. Its efficient execution relies on multidirectional acceleration, deceleration, and control of the supporting leg. Athletes with poor zigzag running performance were more prone to non-contact knee and ankle injuries (31). Therefore, zigzag running serves as a tool for assessing lower-limb kinetic changes in this study. The zigzag running test consisted of a course with four 5-meter segments and a 100° turning angle (Figure 3). Participants started from a standing position at the starting line, sprinted through the course as fast as possible, and were instructed to plant their outside foot and sharply cut around each cone. The sEMG signals were recorded throughout the entire process.

**Figure 3:**
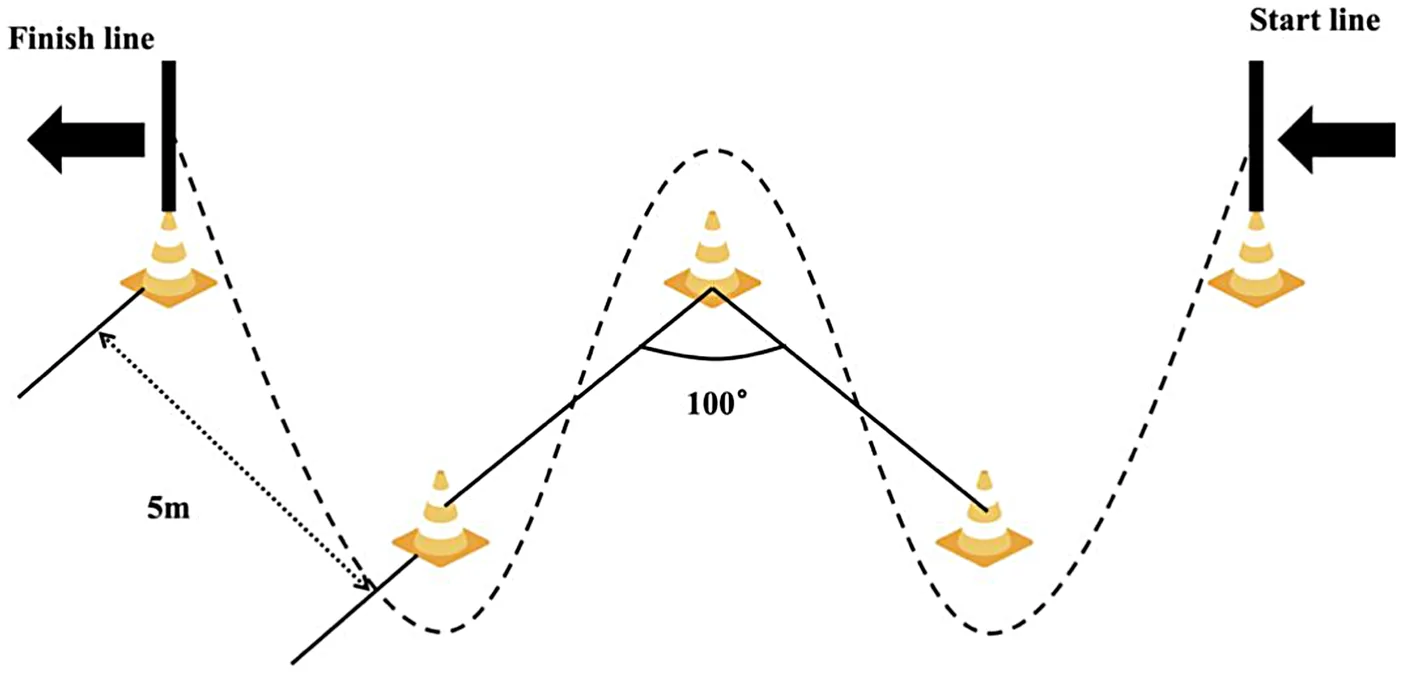
Zigzag running diagram.

### Experimental procedure

Prior to testing, the participants performed 10 min of jogging and stretching, including 5 minutes of static and dynamic stretching for lower limb muscles, followed by 5 min of jogging. They then completed familiarization trials for each task (SLL, DLL, and zigzag running) until they felt comfortable with the procedures. Familiarization was followed immediately by formal testing (within approximately 2 min).

During our testing, to control potential influence of hand position on landing and neuromuscular mechanics, participants maintained forward horizontal gaze and kept hands on hips throughout all the landing tests. And one-minute intervals between the tests to maintain the fatigued state while allowing for a minimal and standardized recovery for equipment reconfiguration. All landing tests were video-recorded (cameras positioned 2 m directly in front and at a 90-degree angle) for qualitative observation and to confirm the validity of each trial (e.g., proper hand placement on hips, successful balance maintenance). Zigzag running was similarly video-recorded at each change-of-direction position.

Tests were performed immediately after warm-up. Firstly, SLL was performed (3 repetitions per side with 20-second intervals between sets). Participants jumped from a 30 cm height, landing on one leg. Then, DLL (3 repetitions, 20-second rest) was performed: Athletes performed a two-legged landing from a 30 cm height. A trial was repeated if the participant failed to meet the acceptance criteria (loss of balance, hands off hips, or incomplete force plate contact); only the 3 valid trials per condition were retained for analysis. No rejected trials were included in the final dataset. The movement trajectory during lower-body landing was recorded, and electromyographic data were collected. Finally, zigzag-running was performed. The kinetic data was collected during landing and the neuromuscular data (sEMG) was collected during the landing and zigzag running.

After completing the above tests, fatigue induction was conducted using the Yo-YoIE2 until the termination criteria were met (failure to reach the line twice upon the audio signal). Post-fatigue testing began immediately after Yo-Yo IE2 completion: SLL was performed within 0–2 min, DLL within 3–5 min, and zigzag running within 6–8 min post-fatigue (Figure 4).

**Figure 4:**
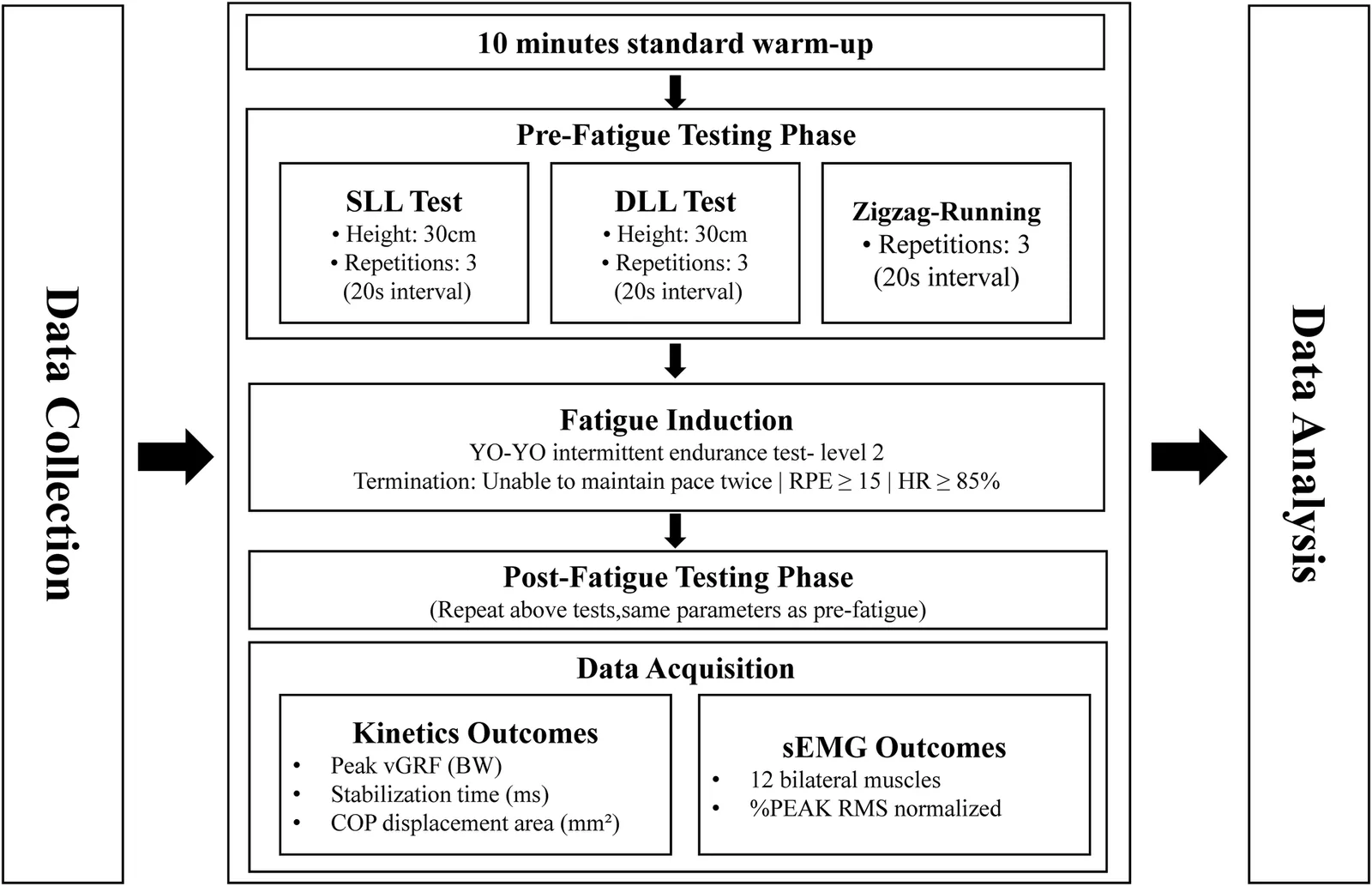
Experimental flowchart.

### Instrumentation and data acquisition

During the SLL and DLL tests, landing stability time, peak vGRF (normalized to body weight, BW) in both feet, and center of pressure (COP) parameters were collected using a Kinvent force plate (Kinvent, Montpellier, France). Simultaneously, muscle activity was recorded using a Cometa sEMG system (Myon Rs sEMG, Cometa, Italy). Signals were recorded from 12 muscles in the left and right legs: rectus femoris, vastus lateralis, vastus medialis, biceps femoris, semitendinosus, and tibialis anterior.

### Data processing and analysis

Raw sEMG signals were sampled at 2,000 Hz. The data underwent rigorous preprocessing following standardized protocols using EMG and Motion Tools software (Cometa S.r.l., Italy). Preprocessing included baseline correction and a cascade of three 4th-order Butterworth filters: a high-pass filter with a 20 Hz cutoff frequency, a low-pass filter with a 100 Hz cutoff frequency, and a notch filter (40–60 Hz) to remove power line interference. The signals were then full-wave rectified and smoothed using a sliding-window root-mean-square (RMS) algorithm with a window size of 200 samples (equivalent to 100 ms). Signals were subsequently normalized using the task-specific peak RMS amplitude recorded during the respective experimental tasks for each muscle. To accurately reflect that true isolated isometric maximum voluntary contractions (MVC) were not measured, this normalized value is expressed as %PEAK. The normalization reference was condition- and task-specific (e.g., pre-fatigue SLL, post-fatigue DLL). For each specific task condition, the %PEAK was calculated using the mean RMS of the three trials as the numerator, and the mean of the task-specific peak RMS values obtained during those exact same three trials as the denominator (%PEAK = Mean RMS of 3 trials/Mean peak RMS of 3 trials × 100%). This task-specific normalization approach follows established practices in sports biomechanics research, where normalizing to peak activation during the actual dynamic task provides functionally relevant information about relative muscle coordination patterns (32, 33). It is important to note that %PEAK reflects relative contribution patterns within each condition, not absolute activation magnitudes. Therefore, changes in %PEAK between pre- and post-fatigue should be interpreted as changes in relative contribution patterns, not as changes in absolute neural drive. This approach is appropriate for our research question, which focuses on comparing muscle coordination strategies within each condition rather than quantifying absolute activation levels. This task-specific normalization approach was adopted because the maximum activation during sport-specific movements (landing and zigzag running) was considered more functionally relevant than isolated isometric MVC testing for the purposes of this study (32, 34, 35).

Force plate data were sampled at 1,000 Hz and filtered using a 2th-order low-pass Butterworth filter with a 50 Hz cutoff frequency. Landing onset was defined as the 5 times the standard deviation of the signal under no-load conditions. Peak vGRF was identified as the maximum vertical force during the landing phase and normalized to body weight: vGRF (BW) = peak vertical force (kg)/body mass (kg). As our force plate outputs data in kilograms, this calculation directly yields a dimensionless body weight ratio (BW). The same value expressed in N/kg would require multiplication by gravitational acceleration (9.81 m/s²). However, all results are reported in BW units throughout the manuscript. Stabilization time was calculated as the duration from initial ground contact until ground reaction force fluctuations fall within ±5% of the subject's body weight. COP displacement area was quantified as the area of the 95% confidence ellipse. For each condition (SLL left, SLL right, DLL), three valid trials were averaged for each participant.

All data processing and analysis were performed using SPSS (Version 27.0; IBM Corp., Armonk, NY, USA). We performed a within-participant design, comparing pre- and post-fatigue measures and left-right asymmetries. Normality of the data was assessed using the Shapiro–Wilk test. For variables with normal distribution, between-condition and between-side comparisons were performed using paired-sample *t*-tests, with results reported as mean differences with 95% confidence intervals (CIs) and Cohen's d effect sizes. For non-normally distributed data, the Wilcoxon signed-rank test was used. No outliers were excluded, and missing data were handled by pairwise deletion. Given the exploratory nature of this study and the relatively small sample size, we did not apply multiple comparison corrections (e.g., Bonferroni or FDR) to avoid inflating the Type II error rate and potentially masking meaningful physiological signals (36). However, as a sensitivity analysis, we performed a *post-hoc* Benjamini-Hochberg False Discovery Rate (FDR) correction (Q = 0.05) for each analysis family defined by task and comparison type: R-SLL (m = 12), L-SLL (m = 12), DLL (m = 12), zigzag (m = 12), landing left-right asymmetry pre-fatigue (m = 12), landing left-right asymmetry post-fatigue (m = 12), zigzag left-right asymmetry pre-fatigue (m = 6), and zigzag left-right asymmetry post-fatigue (m = 6). For kinetic variables: SLL pre- and post-left limb (m = 3), SLL pre- and post-right limb (m = 3), DLL pre- and post-left limb (m = 3), DLL pre- and post-right limb (m = 3), pre- and post-SLL asymmetry (m = 3 each), pre- and post-DLL asymmetry (m = 2 each). The FDR-adjusted p value was calculated as q(k) = p(k) × m/k, with monotonicity enforced. Therefore, findings with uncorrected *P* < 0.05 but FDR *p* > 0.05 are interpreted as exploratory and hypothesis-generating, requiring confirmation in future studies. Statistical significance was set at *P* < 0.05 for uncorrected comparisons.

## Results

A total of 20 subjects met the criteria, with an average age of 20.70 years, average height of 1.68 m, average weight of 58.23 kg, average BMI of 20.69, an average Yo-Yo IE2 distance of 450 m, and an average training duration of 8.10 years (Table 1). All subjects were right-legged. All participants met the predefined fatigue criteria (RPE ≥ 15) prior to post-fatigue testing.

**Table 1:** Basic information of the participants.

Variable	Maximum	Minimum	Values (*n* = 20)
Age (years)	23.00	19.00	20.70 ± 0.98
Height (m)	1.83	1.60	1.68 ± 0.06
Weight (kg)	73.00	45.80	58.23 ± 6.64
BMI (kg/m²)	24.13	17.24	20.69 ± 1.81
RPE	18.00	15.00	16.15 ± 0.81
Years of training (years)	11.00	2.00	8.10 ± 3.02
Weekly training frequency (days)	6.00	3.00	3.75 ± 1.07
Yo-Yo IE2 distance (m)	640	320	450 ± 112.30
Field position (*n*)	—	—	Defender:8, Midfielder:6, Forward:4, Goalkeeper: 2

Values are mean ± standard deviation. BMI, body mass index. Calculation formula: weight/(height × height). All subjects were right-legged. RPE, Rating of Perceived Exertion (Borg 6-20 scale).

In the right-foot single-leg landing (R-SLL) task, the left rectus femoris showed a significant increase in sEMG amplitude post-fatigue [pre: 7.82 (7.45, 8.75) vs. post: 8.64 (7.42, 11.37), median (IQR), Z = −2.016, r = 0.45, *P* = 0.044; exploratory, FDR-adjusted *P* > 0.05]. During left-foot single-leg landing (L-SLL), the left biceps femoris (L.Biceps Fem) also showed a significant increase post-fatigue (mean difference = −1.19, 95% CI: −2.31 to −0.64, *P* = 0.039; exploratory, FDR-adjusted *P* > 0.05) No other significant changes were found in the SLL task. During the DLL task, post-fatigue sEMG amplitude was significantly lower in the right vastus lateralis (R.Vlo) [pre: 8.58 (6.94, 11.55) vs. post: 7.78 (6.50, 10.47), median (IQR), Z = −3.136, r = 0.70, *P* = 0.002; FDR-adjusted *P* = 0.024], right vastus medialis (R.Vmo) [pre: 9.86 (7.64, 13.14) vs. post: 8.27 (6.94, 10.71), Z = −2.576, r = 0.58, *P* = 0.010; FDR-adjusted *P* = 0.040], and left vastus lateralis (L.Vlo) [pre: 9.45 (7.59, 11.02) vs. post: 8.36 (6.44, 10.22), Z = −2.725, r = 0.61, *P* = 0.006; FDR-adjusted *P* = 0.036]. In zigzag running, no significant differences were observed in any muscle before and after fatigue induction (*P* > 0.05) (Tables 2,3).

**Table 2:** Differences in muscle function before and after fatigue during SLL task.

Muscle	R-SLL					L-SLL						
	Pre-fatigue	Post-fatigue	*p*	FDR-Adjusted p	Cohen's d	|r|	Pre-fatigue	Post-fatigue	*p*	FDR-Adjusted p	Cohen's d	|r|
R.Rectus Fem.	7.05 (6.07, 9.56)	6.93 (6.07, 8.47)	0.351	0.574	–	0.21	7.56 (7.16, 9.32)	8.57 (7.04, 10.06)	0.073	0.438	–	0.40
R.Vlo	8.39 ± 2.18	7.92 ± 2.05	0.219	0.574	0.28	–	7.24 ± 1.54	7.82 ± 1.58	0.236	0.444	−0.27	–
R.Vmo	7.95 (6.60, 10.37)	7.49 (6.00, 9.46)	0.455	0.574	–	0.17	7.25 (6.43, 9.26)	7.24 (6.64, 8.16)	0.313	0.444	–	0.23
R.Biceps Fem.	10.36 (9.34, 12.12)	10.68 (8.99, 12.89)	0.411	0.574	–	0.18	9.63 ± 1.78	9.05 ± 2.32	0.348	0.444	0.22	–
R.Semitend.	10.43 ± 2.51	10.71 ± 2.72	0.718	0.783	−0.11	–	8.70 (6.93, 11.96)	9.58 (7.88, 11.29)	0.351	0.444	–	0.21
R.Tib.Ant.	10.12 ± 2.41	10.64 ± 2.09	0.426	0.574	−0.18	–	8.97 ± 1.83	9.59 ± 2.03	0.291	0.444	−0.24	–
L.Rectus Fem.	7.82 (7.45, 8.75)	8.64 (7.42, 11.37)	**0** **.** **044**	0.366	–	0.45	7.35 (6.45, 9.55)	7.02 (6.17, 10.73)	0.881	0.881	–	0.03
L.Vlo	7.61 (6.54, 8.60)	6.85 (6.10, 9.28)	0.433	0.574	–	0.18	7.84 (6.46, 10.13)	7.14 (6.32, 10.16)	0.117	0.444	–	0.35
L.Vmo	7.46 (6.44, 9.55)	7.69 (6.86, 9.92)	0.478	0.574	–	0.16	7.72 (6.55, 11.01)	7.64 (6.00, 9.51)	0.370	0.444	–	0.20
L.Biceps Fem.	9.72 ± 2.08	10.09 ± 2.20	0.388	0.574	−0.20	–	10.18 ± 2.39	11.37 ± 2.97	**0**.**039**	0.438	−0.50	–
L.Semitend.	9.94 ± 2.10	11.27 ± 2.66	0.061	0.366	−0.45	–	10.54 ± 2.48	11.32 ± 2.42	0.197	0.444	−0.30	–
L.Tib.Ant.	9.91 ± 1.50	10.02 ± 2.51	0.807	0.807	−0.55	–	9.08 (8.02, 11.57)	9.18 (8.11, 10.60)	0.765	0.835	–	0.07

Values are presented as Mean ± SD for normally distributed variables (analyzed by paired *t*-test; effect size: Cohen's d) or as Median (IQR) for non-normally distributed variables (analyzed by Wilcoxon signed-rank test; effect size: r = |Z|/√N). Rectus Fem: Rectus Femoris, Vlo: Vastus Lateralis, Vmo: Vastus Medialis Obliquus, Biceps Fem: Biceps Femoris, Semitend: Semitendinosus, Tib Ant: Tibialis Anterior. Bold values indicate *P* < 0.05 (uncorrected). *P* values are uncorrected for multiple comparisons; see Statistical Analysis for details. %PEAK = (Mean RMS/Mean Task-specific Peak RMS) × 100%, where Mean Task-specific Peak RMS is the average of task-specific peak RMS values across the three trials for each muscle within a specific task.

**Table 3:** Differences in muscle function before and after fatigue during DLL and zigzag running tasks.

Muscle	DLL						Zigzag running					
	Pre-fatigue	Post-fatigue	*p*	FDR-Adjusted p	Cohen's d	|r|	Pre-fatigue	Post-fatigue	*p*	FDR-Adjusted p	Cohen's d	|r|
R.Rectus Fem.	7.78 (6.37, 10.10)	6.81 (5.96, 10.12)	0.279	0.478	_	0.24	12.33 (12.12, 14.06)	13.27 (12.09, 14.29)	0.296	0.930	_	0.23
R.Vlo	8.58 (6.94, 11.55)	7.78 (6.50, 10.47)	**0** **.** **002**	**0**.**024**	_	0.70	12.23 (10.90, 12.75)	11.85 (10.74, 13.22)	0.852	0.930	_	0.04
R.Vmo	9.86 (7.64, 13.14)	8.27 (6.94, 10.71)	**0**.**010**	**0**.**040**	_	0.58	11.94 ± 1.51	11.91 ± 1.69	0.954	0.954	0.01	_
R.Biceps Fem.	8.82 (7.93, 11.77)	8.04 (7.51, 10.51)	0.737	0.737	_	0.08	14.75 ± 2.35	14.30 ± 3.13	0.542	0.930	0.14	_
R.Semitend.	8.07 (6.43, 8.93)	7.75 (6.29, 9.42)	0.478	0.521	_	0.33	14.37 (13.26, 15.20)	14.23 (12.82, 15.14)	0.478	0.930	_	0.16
R.Tib.Ant.	9.00 ± 3.14	8.44 ± 2.94	0.243	0.478	0.27	_	14.41 ± 1.77	14.29 ± 1.31	0.814	0.930	0.05	-
L.Rectus Fem.	8.21 (6.68, 10.28)	7.62 (6.06, 9.21)	0.332	0.493	_	0.22	12.01 (11.50, 12.93)	12.45 (11.13, 13.29)	0.601	0.930	_	0.12
L.Vlo	9.45 (7.59, 11.02)	8.36 (6.44, 10.22)	**0**.**006**	**0**.**036**	_	0.61	11.52 (11.37, 12.78)	11.69 (10.68, 12.63)	0.502	0.930	_	0.19
L.Vmo	9.73 (7.84, 12.55)	8.95 (7.16, 10.70)	0.218	0.478	_	0.28	12.13 ± 1.25	12.03 ± 1.09	0.820	0.930	0.05	_
L.Biceps Fem.	8.15 (7.20, 9.96)	9.52 (7.26, 11.50)	0.370	0.493	_	0.20	14.72 ± 2.17	14.06 ± 1.48	0.303	0.930	0.24	_
L.Semitend.	8.97 (6.90, 10.20)	7.97 (6.70, 11.20)	0.455	0.521	_	0.17	13.98 ± 1.72	14.16 ± 2.05	0.675	0.930	−0.10	_
L.Tib.Ant.	9.57 ± 2.74	9.10 ± 2.42	0.184	0.478	0.31	_	15.36 ± 2.07	14.84 ± 2.63	0.269	0.930	0.26	_

Values are presented as Mean ± SD for normally distributed variables (analyzed by paired *t*-test; effect size: Cohen's d) or as Median (IQR) for non-normally distributed variables (analyzed by Wilcoxon signed-rank test; effect size: r = |Z|/√N). Rectus Fem: Rectus Femoris, Vlo: Vastus Lateralis, Vmo: Vastus Medialis Obliquus, Biceps Fem: Biceps Femoris, Semitend: Semitendinosus, Tib Ant: Tibialis Anterior. Bold values indicate *P* < 0.05 (uncorrected). *P* values are uncorrected for multiple comparisons; see Statistical Analysis for details. %PEAK = (Mean RMS/Mean Task-specific Peak RMS) × 100%, where Mean Task-specific Peak RMS is the average of task-specific peak RMS values across the three trials for each muscle within a specific task.

In the SLL, no significant differences were observed between the left and right landing legs before and after fatigue induction (*P* > 0.05). Similarly, no significant asymmetry in sEMG amplitude was found between sides in DLL before and after fatigue induction (*P* > 0.05). In the zigzag running, the left rectus femoris showed significantly lower sEMG amplitude than the right side before fatigue [left: 12.01 (11.50, 12.93) vs. right: 12.33 (12.12, 14.06), median (IQR), Z = −2.091, r = 0.47, *P* = 0.037; exploratory, FDR-adjusted *P* > 0.05], whereas no significant side-to-side difference was observed after fatigue (*P* = 0.112). To directly test whether this asymmetry significantly decreased, we compared pre- and post-fatigue bilateral difference values (right−left) using a paired *t*-test. The mean difference changed from 1.248 ± 2.376%PEAK pre-fatigue to 0.915 ± 2.456%PEAK post-fatigue, but this reduction was not significant (mean reduction=0.334, 95% CI: −1.16–1.83, *P* = 0.645) (Tables 4,5).

**Table 4:** Differences in left-right leg before and after fatigue during landing tasks.

Muscle name		SLL						DLL					
		Left	Right	*p*	FDR-Adjusted p	Cohen's d	|r|	Left	Right	*p*	FDR-Adjusted p	Cohen's d	|r|
Pre-fatigue	Rectus Fem.	7.35 (6.45, 9.55)	7.05 (6.07, 9.56)	0.478	0.701	_	0.16	8.22 (6.68, 10.28)	7.78 (6.37, 10.10)	0.940	0.940	_	0.02
	Vlo	7.84 (6.46, 10.31)	8.44 (6.21, 10.47)	0.526	0.701	_	0.14	9.45 (7.59, 11.02)	8.58 (6.94, 11.55)	0.332	0.701	_	0.22
	Vmo	7.72 (6.55, 11.01)	7.95 (6.60, 10.37)	0.370	0.701	_	0.20	9.73 (7.84, 12.55)	9.86 (7.64, 13.14)	0.433	0.701	_	0.18
	Biceps Fem.	9.98 (8.62, 11.20)	10.36 (9.34, 12.12)	0.145	0.701	_	0.33	8.15 (7.20, 9.96)	8.82 (7.93, 11.77)	0.627	0.752	_	0.11
	Semitend.	10.54 ± 2.48	10.43 ± 2.51	0.831	0.907	0.05	_	8.97 (6.90, 10.20)	8.07 (6.43, 8.93)	0.179	0.701	_	0.30
	Tib.Ant.	9.55 ± 2.26	10.12 ± 2.41	0.341	0.701	−0.22	_	9.57 ± 2.74	9.00 ± 3.14	0.117	0.701	0.37	_
Post-fatigue	Rectus Fem.	7.02 (6.17, 10.73)	6.93 (6.07, 8.47)	0.167	0.634	_	0.31	7.62 (6.06, 9.21)	6.82 (5.96, 10.12)	0.502	0.693	_	0.15
	Vlo	7.15 (6.32, 10.16)	7.50 (6.24, 9.29)	0.881	0.960	_	0.03	8.57 ± 2.50	8.55 ± 2.50	0.960	0.960	0.01	_
	Vmo	7.64 (6.00, 9.51)	7.49 (6.00, 9.46)	0.332	0.634	_	0.22	8.95 (7.16, 10.70)	8.27 (6.94, 10.71)	0.370	0.634	_	0.20
	Biceps Fem.	11.37 ± 2.97	10.77 ± 2.55	0.520	0.693	0.15	_	9.52 (7.26, 11.50)	8.04 (7.51, 10.51)	0.654	0.785	_	0.10
	Semitend.	11.32 ± 2.42	10.71 ± 2.72	0.295	0.634	0.24	_	7.97 (6.70, 11.20)	7.75 (6.29, 9.42)	0.313	0.634	_	0.23
	Tib.Ant.	9.18 (8.11, 10.60)	10.34 (9.37, 12.11)	0.179	0.634	_	0.30	9.10 ± 2.42	8.44 ± 2.94	0.162	0.634	0.33	_

Values are presented as Mean ± SD for normally distributed variables (analyzed by paired *t*-test; effect size: Cohen's d) or as Median (IQR) for non-normally distributed variables (analyzed by Wilcoxon signed-rank test; effect size: r = |Z|/√N). Rectus Fem: Rectus Femoris, Vlo: Vastus Lateralis, Vmo: Vastus Medialis Obliquus, Biceps Fem: Biceps Femoris, Semitend: Semitendinosus, Tib Ant: Tibialis Anterior. Bold values indicate *P* < 0.05 (uncorrected). *P* values are uncorrected for multiple comparisons; see Statistical Analysis for details. %PEAK = (Mean RMS/Mean Task-specific Peak RMS) × 100%, where Mean Task-specific Peak RMS is the average of task-specific peak RMS values across the three trials for each muscle within a specific task.

**Table 5:** Differences in left-right leg before and after fatigue during zigzag running.

Muscle name		Zigzag running					
		Left	Right	*p*	FDR-Adjusted *p*	Cohen's d	|r|
Pre-fatigue	Rectus Fem.	12.01 (11.50, 12.93)	12.33 (12.12, 14.06)	**0** **.** **037**	0.222	_	0.47
	Vlo	11.53 (11.37, 12.78)	12.23 (10.90, 12.75)	0.709	0.851	_	0.08
	Vmo	12.13 ± 1.25	11.94 ± 1.51	0.611	0.851	0.12	_
	Biceps Fem.	14.72 ± 2.17	14.75 ± 2.35	0.974	0.974	−0.01	_
	Semitend.	13.98 ± 1.72	14.58 ± 1.94	0.306	0.612	−0.24	_
	Tib.Ant.	15.36 ± 2.07	14.41 ± 1.77	0.132	0.396	0.35	_
Post-fatigue	Rectus Fem.	12.32 ± 1.65	13.24 ± 1.66	0.112	0.672	−0.37	_
	Vlo	11.69 (10.68, 12.63)	11.85 (10.74, 13.22)	0.313	0.773	_	0.23
	Vmo	12.03 ± 1.09	11.91 ± 1.69	0.756	0.773	0.07	_
	Biceps Fem.	13.98 (13.25, 14.94)	14.03 (12.26, 15.66)	0.773	0.773	_	0.13
	Semitend.	14.16 ± 2.05	13.96 ± 2.26	0.750	0.773	0.07	_
	Tib.Ant.	14.84 ± 2.63	14.29 ± 1.31	0.388	0.773	0.20	_

Values are presented as Mean ± SD for normally distributed variables (analyzed by paired *t*-test; effect size: Cohen's d) or as Median (IQR) for non-normally distributed variables (analyzed by Wilcoxon signed-rank test; effect size: r = |Z|/√N). Rectus Fem: Rectus Femoris, Vlo: Vastus Lateralis, Vmo: Vastus Medialis Obliquus, Biceps Fem: Biceps Femoris, Semitend: Semitendinosus, Tib Ant: Tibialis Anterior. Bold values indicate *P* < 0.05 (uncorrected). *P* values are uncorrected for multiple comparisons; see Statistical Analysis for details. %PEAK = (Mean RMS/Mean Task-specific Peak RMS) × 100%, where Mean Task-specific Peak RMS is the average of task-specific peak RMS values across the three trials for each muscle within a specific task.

In the SLL, peak vertical ground reaction force (vGRF, in BW) significantly decreased after fatigue induction (left foot: 2.52 ± 0.31 BW vs. 2.34 ± 0.27 BW, *P* = 0.015, FDR-adjusted *P* = 0.045; right foot: 2.69 ± 0.36 BW vs. 2.40 ± 0.30 BW, *P* < 0.001, FDR-adjusted *P* < 0.003). Left foot stability time significantly increased after fatigue induction [722.25 ± 282.58 ms vs. 765.50 (573.75, 1290.75) ms, *P* = 0.037, FDR-adjusted *P* = 0.056, exploratory]. COP displacement area showed no significant differences before and after fatigue induction. In DLL, only right foot peak vGRF (BW) decreased significantly after fatigue induction (1.52 ± 0.37 BW vs. 1.40 ± 0.32 BW, *P* = 0.048, FDR-adjusted *P* = 0.144, exploratory), with no significant differences observed in other variables (Figures 5a,c,e and Table 6).

**Figure 5:**
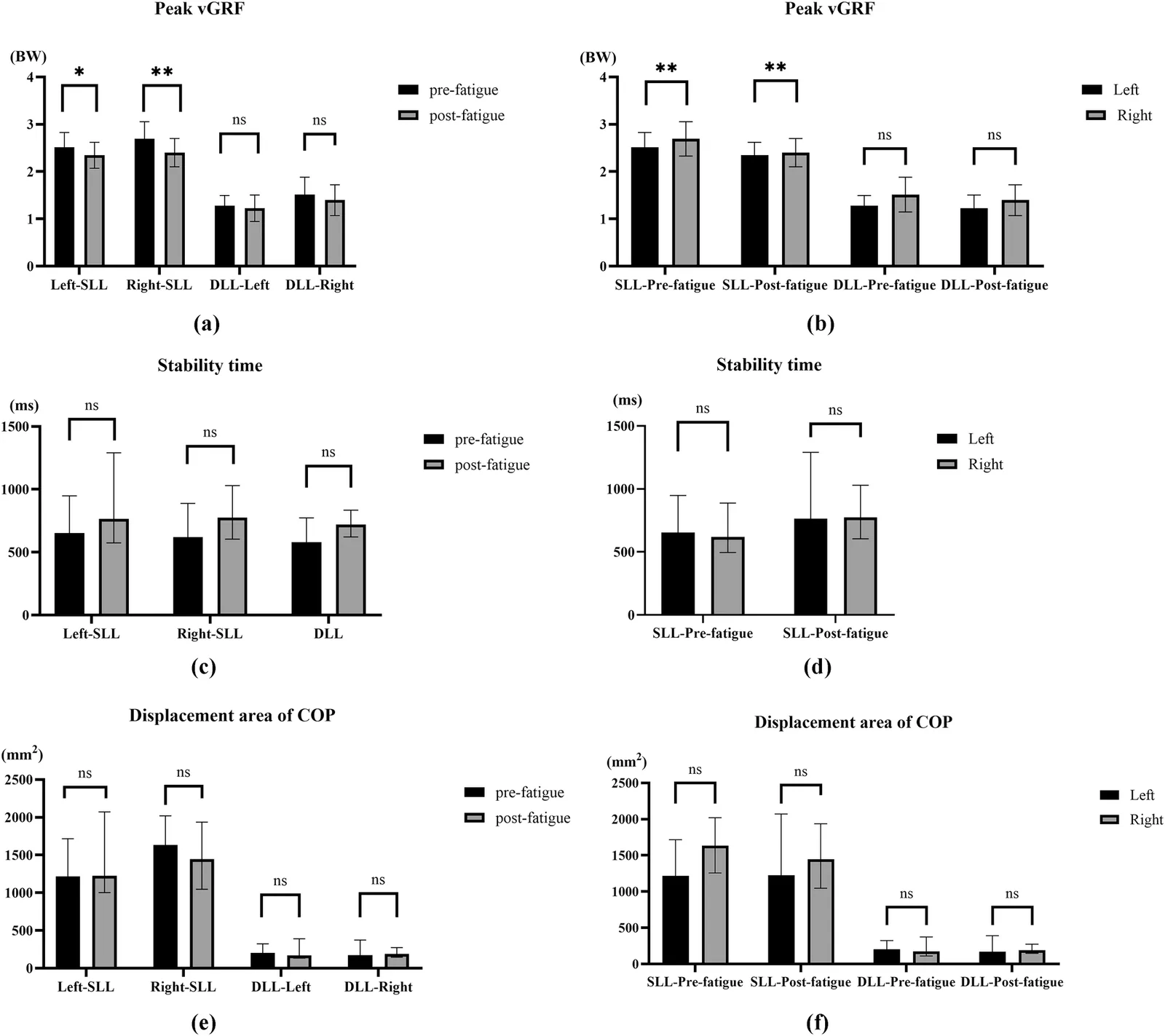
Comparisons of kinetic variables before and after fatigue, and bilateral asymmetry. **(a)** Peak vGRF pre- vs. post-fatigue; **(b)** left-right asymmetry in peak vGRF; **(c)** stabilization time pre- vs. post-fatigue; **(d)** left-right asymmetry in stabilization time; **(e)** COP displacement area pre- vs. post-fatigue; **(f)** left-right asymmetry in COP displacement area. Peak vGRF is expressed in body weight (BW). For charts based entirely on a normal distribution, the bar height represents the mean (Mean), and the error bars represent the standard deviation (SD). For charts that include non-normal distributions, the bar height represents the median (IQR), and the error bars represent the upper and lower quartiles. Significance markers (*p < 0.05, **p < 0.01, ***p < 0.001) indicate comparisons that remained significant after Benjamini-Hochberg FDR correction (Q = 0.05). Non-significant comparisons (ns) include both originally non-significant results and those that did not survive FDR correction. All FDR-adjusted *P* values are reported in Tables 6,7.

**Table 6:** Differences in dynamic parameters before and after fatigue.

Task	Index	Left						Right					
		Pre-fatigue	Post-fatigue	*p*	FDR-Adjusted *P*	Cohen's d	|r|	Pre-fatigue	Post-fatigue	*p*	FDR-Adjusted *P*	Cohen's d	|r|
SLL	vGRF (BW)	2.52 ± 0.31	2.34 ± 0.27	**0** **.** **015**	**0**.**045**	0.596	–	2.69 ± 0.36	2.40 ± 0.30	**<0**.**001**	**<0**.**003**	1.040	–
	stability time (ms)	722.25 ± 282.58	765.50 (573.75, 1,290.75)	**0**.**037**	0.056	–	0.468	619.00 (496.00, 888.00)	774.50 (604.25, 1,028.75)	0.126	0.189	–	0.342
	COP (㎟)	1,354.80 ± 652.58	1,223.00 (1,004.00, 2,072.25)	0.135	0.135	–	0.334	1,689.25 ± 653.65	1,445.00 (1047.75, 1,937.50)	0.681	0.681	–	0.092
DLL	vGRF (BW)	1.21 (1.10, 1.50)	1.22 ± 0.28	0.173	0.260	–	0.305	1.52 ± 0.37	1.40 ± 0.32	**0**.**048**	0.144	0.472	–
	stability time (ms)	635.65 ± 213.42	719.00 (622.25, 833.75)	0.121	0.260	–	0.346	635.65 ± 213.42	719.00 (622.25, 833.75)	0.121	0.182	–	0.346
	COP (㎟)	206.00 (100.78, 324.25)	169.00 (133.50, 391.00)	0.879	0.879	–	0.034	174.00 (111.50, 374.00)	191.00 (147.00, 273.75)	0.747	0.747	–	0.072

Values are presented as Mean ± SD for normally distributed variables (analyzed by paired *t*-test; effect size: Cohen's d) or as Median (IQR) for non-normally distributed variables (analyzed by Wilcoxon signed-rank test; effect size: r = |Z|/√N). SLL, single-leg landing; DLL, double-leg landing; vGRF, vertical ground reaction force; COP, center of pressure. Bold values indicate *P* < 0.05 (uncorrected). FDR-adjusted *P* values were calculated using the Benjamini-Hochberg procedure (Q = 0.05) within each analysis family: SLL left limb (m = 3), SLL right limb (m = 3), DLL left limb (m = 3), DLL right limb (m = 3). Bold FDR-adjusted *P* values indicate statistical significance after correction.

**Table 7:** Asymmetry in biomechanical parameters before and after fatigue.

Task	Index	Pre-fatigue						Post-fatigue					
		Left	Right	*p*	FDR-Adjusted *P*	Cohen's d	|r|	Left	Right	*p*	FDR-Adjusted *P*	Cohen's d	|r|
SLL	vGRF(BW)	2.52 ± 0.31	2.69 ± 0.36	**0**.**002**	**0**.**006**	−0.827	–	2.34 ± 0.27	2.40 ± 0.30	**<0**.**01**	**<0**.**03**	−0.252	–
	stability time (ms)	722.25 ± 282.58	619.00 (496.00, 888.00)	0.911	0.911	–	0.025	765.50 (573.75, 1,290.75)	774.50 (604.25, 1,028.75)	0.478	0.478	–	0.159
	COP (㎟)	1,354.80 ± 652.58	1,689.25 ± 653.65	0.636	0.911	−0.384	–	1,223.00 (1,004.00, 2,072.25)	1,445.00 (1,047.75, 1,937.50)	0.433	0.478	–	0.175
DLL	vGRF (BW)	1.21 (1.10, 1.50)	1.52 ± 0.37	0.050	0.100	–	0.438	1.22 ± 0.28	1.40 ± 0.32	0.424	0.848	−0.370	–
	COP (㎟)	206.00 (100.78, 324.25)	174.00 (111.50, 374.00)	0.737	0.737	–	0.075	169.00 (133.50, 391.00)	191.00 (147.00, 273.75)	0.852	0.852	–	0.042

Values are presented as Mean ± SD for normally distributed variables (analyzed by paired *t*-test; effect size: Cohen's d) or as Median (IQR) for non-normally distributed variables (analyzed by Wilcoxon signed-rank test; effect size: r = |Z|/√N). SLL, single-leg landing; DLL, double-leg landing; vGRF: vertical ground reaction force; COP, center of pressure. Bold values indicate *P* < 0.05 (uncorrected). FDR-adjusted *P* values were calculated using the Benjamini-Hochberg procedure (Q = 0.05) within each analysis family: SLL pre-fatigue (m = 3), SLL post-fatigue (m = 3), DLL pre-fatigue (m = 2), DLL post-fatigue (m = 2). Bold FDR-adjusted *P* values indicate statistical significance after correction. BW, body weight.

Before fatigue induction, significant differences existed in peak vGRF (BW) between the left and right feet in the SLL (left foot 2.52 ± 0.31 BW vs. right foot 2.69 ± 0.36 BW, *P* = 0.002, FDR-adjusted *P* = 0.006). After fatigue, the significant differences in peak vGRF (BW) between the left and right feet still existed (left foot 2.34 ± 0.27 BW vs. right foot 2.40 ± 0.30 BW, *P* < 0.01, FDR-adjusted *P* < 0.03). peak vGRF (BW) in DLL, stabilization time and COP displacement area showed no significant lateral asymmetry before or after fatigue induction (Figures 5b,d,e and Table 7).

## Discussion

In this study, fatigue was associated with task-specific neuromuscular and kinetic changes, though the fixed testing order and potential recovery may have influenced the observed patterns. In the SLL task, L.Rectus Femoris (R-SLL) and L.Biceps Femoris (L-SLL) showed significant increases in normalized sEMG amplitude post-fatigue. In the DLL task, R.Vlo, L.Vlo, and R.Vmo showed significant decreases in normalized sEMG amplitude. In zigzag running, the pre-fatigue bilateral asymmetry (*P* = 0.037) was no longer significant after fatigue (*P* = 0.112). However, as zigzag was performed last (6–8 min post-Yo-Yo), partial recovery may partially explain this absence. Peak vGRF decreased bilaterally in SLL and in the right foot during DLL, while stabilization time prolonged in SLL. These findings indicate task-specific trends following fatigue, though causal conclusions regarding task dependency are limited by the testing sequence.

Previous studies have shown that fatigue alters lower limb muscle activation patterns (10). We observed task-specific trends in our data; however, the fixed testing sequence and potential partial recovery (particularly for zigzag running) limit attribution of these patterns solely to fatigue. The body compensates for diminished overall control by selectively enhancing activation in specific muscle groups, aligning with the past studies that fatigue may induce protective compensatory adaptations (10). For example, this study showed an exploratory increase in left biceps femoris normalized sEMG amplitude during the L-SLL task (*P* = 0.039; FDR-adjusted *P* > 0.05), which is directionally consistent with past studies of increased biceps femoris activation following single-leg landing fatigue (8), though this finding requires confirmation.

Our study showed that the normalized sEMG amplitude of biceps femoris appeared higher than that of semitendinosus during landing in our collegiate cohort; however, due to the task-specific normalization method, this observation reflects relative patterns within each condition and should not be interpreted as evidence of absolute differences in neural drive between muscles. Therefore, we acknowledge that this observation is tentative. This contrasts with Strutzenberger et al., who reported medial hamstring dominance in elite female soccer players during fatigued squat landings (37). This discrepancy may reflect differences in task demands (drop landing vs. squat landing), athlete levels (collegiate vs. elite), and associated neuromuscular adaptation strategies (20). Notably, we did not directly measure training status, so this interpretation remains speculative. A lateral-dominant pattern during unilateral landing could serve a protective role by resisting knee valgus and rotational forces (9), though this requires confirmation through kinematic analysis. Furthermore, muscle activation amplitude alone does not directly determine ACL injury risk, as joint kinematics and loading rates are equally critical (9).

Significant left-right asymmetry in the rectus femoris was found during zigzag running before fatigue induction (*P* = 0.037; exploratory, FDR-adjusted *P* > 0.05), but was no longer statistically significant after fatigue (*P* = 0.112). Some studies have noted that zigzag running involves frequent abrupt stops and turns, increasing knee varus and shear forces, and may lead to reduced coordination, exacerbating lower limb asymmetry, thereby substantially elevating the risk of acute non-contact injuries (38, 39). While the present study showed a trend towards reduced asymmetry, the exploratory nature of this finding precludes strong conclusions. The apparent reduction may reflect natural variability rather than a true neuromuscular adaptation.

Additionally, participants in this study exhibited a noticeable decrease in sEMG amplitude after fatigue induction. The reduction in lateral differences observed in this study could be interpreted in multiple ways. While it may reflect a strategic shift towards more symmetrical control, it could also indicate bilateral neuromuscular fatigue or reduced neural drive to both limbs. Without concurrent kinematic data, we cannot determine whether this pattern represents an adaptive strategy or a maladaptive response. This interpretation therefore remains speculative and requires further investigation incorporating joint angle and moment analyses. Alternatively, the timing of DLL (3–5 min) versus zigzag (6–8 min) post-Yo-Yo may have influenced the observed patterns across tasks. This also aligns with past studies that bilateral muscle load sharing during double-leg landing resulted in a more efficient energy dissipation strategy (40). Although there was a pre-fatigue trend for the dominant leg to absorb a higher load during DLL (pre-fatigue left vs. right *P* = 0.050), this asymmetry was not observed post-fatigue (*P* = 0.424). This suggests a more balanced distribution of landing forces between the limbs following fatigue.

Regarding lower-limb kinetic performance, peak vGRF (BW) in both feet significantly decreased after fatigue induction during the SLL test (left foot: pre 2.52 ± 0.31 BW vs. post 2.34 ± 0.27 BW, mean difference = −0.18 BW, *P* = 0.015, FDR-adjusted *P* = 0.045; right foot: pre 2.69 ± 0.36 BW vs. post 2.40 ± 0.30 BW, mean difference = −0.29 BW, *P* < 0.001, FDR-adjusted *P* < 0.003). The discrepancy between our findings and some previous studies (40) that reported increased vGRF following fatigue could be attributed to several methodological and population-specific factors. First, the specific fatigue protocol (Yo-Yo IE2 vs. other custom protocols) and its intensity and duration may differently affect neuromuscular function and landing mechanics (10). Second, the landing height (30 cm in our study) and the specific task demands can influence loading patterns. Third, the characteristics of the study populations differ. While our participants were collegiate players, other studies often involved elite or professional athletes. Differences in training history (20), baseline strength, and neuromuscular adaptation levels could contribute to the divergent findings. It is also possible that our participants adopted a more conservative landing strategy when fatigued to maintain stability, thereby reducing peak vGRF. We acknowledge that our current dataset does not allow us to conclusively differentiate between these possibilities, and further research controlling for these variables is necessary.

Post-fatigue stabilization time increased (*P* = 0.037; FDR-adjusted *P* = 0.056, exploratory), but COP displacement area showed no significant change before and after fatigue induction. The prolonged stabilization time reflects diminished landing cushioning and balance regulation capacity after fatigue, consistent with past studies among Japanese women's soccer league players that fatigue reduced hip and ankle flexion angles, decreases cushioning efficiency, and consequently extends stabilization time (41), though this finding did not survive FDR correction and should be interpreted as exploratory. The tendency toward bilateral COP control in the SLL test suggested that fatigue may prompt players to adopt more conservative and symmetrical postural control strategies during unilateral support tasks.

Under fatigue, the body may enhance bilateral lower limb coordination to compensate for insufficient unilateral muscle activation or reduced neural control efficiency, thereby maintaining landing stability. Similar phenomena have been reported in other fatigue-related studies. For instance, some studies noted that players increased contralateral limb involvement during unilateral tasks after fatigue to optimize overall postural control (16). Furthermore, other studies observed enhanced symmetry in COP trajectories during unilateral movements after fatigue, suggesting a protective adaptation to mitigate injury risks from control asymmetry (42). Although COP coordination showed an enhancing trend after fatigue, the displacement area did not significantly increase. This indicates that under the experimental conditions, players could still effectively control the amplitude of center-of-pressure fluctuations. We observed no significant change in COP displacement area post-fatigue, despite changes in muscle normalized sEMG amplitude. However, as we did not perform a formal correlation analysis, no relationship between these variables can be inferred. Changes in COP cannot be fully explained by electromyographic normalized sEMG amplitude levels alone. Future research integrating joint kinematic and kinetic data may further reveal the intrinsic relationship between COP control and lower-limb load distribution during fatigue.

Several limitations should be acknowledged. First, the sample size was relatively small and limited to players from two university teams, restricting generalizability to other populations (e.g., professional athletes). Second, the within-subject pre-post design lacked a non-fatigued control group, which cannot fully account for learning effects or time-related performance changes. Third, the fixed task sequence (SLL to DLL to zigzag running) and short recovery intervals (1 min) between post-fatigue tests may have introduced order effects and allowed partial recovery. Zigzag was performed last (6–8 min post-Yo-Yo), potentially allowing greater recovery than earlier tasks, which limits causal attribution of task-specific patterns to fatigue. Fourth, we did not analyze fatigue × side interaction, which could provide further insights into asymmetry patterns. Besides, while we recorded participants’ field positions, we did not include position as a factor in our analyses. Position-specific neuromuscular responses to fatigue may differ (e.g., goalkeepers vs. outfield players), and future studies with larger samples are needed to address this question. Fifth, methodological constraints include the use of an estimated (220-age) maximum heart rate and the absence of kinematic data to contextualize neuromuscular and kinetic findings and the lack of humidity measurement, although all tests were conducted in a controlled laboratory environment at approximately 25°C. Additionally, sleep, nutrition, and menstrual cycle data were not collected. Finally, no multiple comparison corrections were applied *a priori* for the EMG and kinetic variables—a deliberate choice to minimize Type II errors given the exploratory design, but which increases the risk of Type I errors. As a sensitivity analysis, we performed a *post-hoc* Benjamini-Hochberg FDR correction (Q = 0.05) for each analysis family (R-SLL: m = 12; L-SLL: m = 12; DLL: m = 12; zigzag: m = 12; landing left-right asymmetry pre/post: m = 12 each; zigzag left-right asymmetry pre/post: m = 6 each). For kinetic variables: SLL pre/post left limb (m = 3), SLL pre/post right limb (m = 3), DLL pre/post left limb (m = 3), DLL pre/post right limb (m = 3), SLL asymmetry pre/post (m = 3 each), DLL asymmetry pre/post (m = 2 each). After FDR correction, only three DLL findings remained significant: R.Vlo (FDR *p* = 0.024), L.Vlo (FDR *p* = 0.036), and R.Vmo (FDR *p* = 0.040). All other originally significant results (*P* = 0.037, 0.039, 0.044) did not survive correction (FDR *p* > 0.05). Therefore, all findings except the three DLL vastus muscles should be interpreted as exploratory and preliminary, requiring confirmation in future studies with larger sample sizes and pre-registered hypotheses.

## Conclusion

Soccer-specific fatigue induces task-dependent neuromuscular and kinetic changes in female collegiate players: reduced vastus muscles normalized sEMG amplitude during DLL (with R.Vlo, L.Vlo, and R.Vmo remaining significant after FDR correction), decreased peak vGRFs with prolonged stabilization time during SLL, and exploratory trends towards reduced asymmetry in zigzag running. Given the exploratory nature of most findings, these results require confirmation in future studies with larger sample sizes. Preliminary training implications include emphasis on unilateral stability and multi-directional neuromuscular control. These findings suggest that training programs for collegiate female players should emphasize: (1) unilateral stability and support strength to address the prolonged stabilization time and reduced peak forces observed during SLL; (2) quadriceps fatigue resistance training to counteract the reduced vastus muscles normalized sEMG amplitude during DLL; and (3) multi-directional neuromuscular control to prepare for the demands of zigzag running and similar change-of-direction tasks. These targeted approaches may help mitigate potential injury risks under fatigued conditions.

## Data Availability

The raw data supporting the conclusions of this article will be made available by the authors, without undue reservation.
